# Recombinant human soluble thrombomodulin in patients with sepsis-associated coagulopathy (SCARLET): an updated meta-analysis

**DOI:** 10.1186/s13054-019-2587-2

**Published:** 2019-09-05

**Authors:** Kazuma Yamakawa, Jerrold H. Levy, Toshiaki Iba

**Affiliations:** 1 Division of Trauma and Surgical Critical Care, Osaka General Medical Center, 3-1-56 Bandai-Higashi, Sumiyoshi, Osaka, 558-8558 Japan; 2 Department of Anesthesiology, Critical Care, and Surgery, Duke University School of Medicine, Durham, NC USA; 3 Department of Emergency and Disaster Medicine, Juntendo University Graduate School of Medicine, Tokyo, Japan

Letter to the editor

The reported trial of recombinant human soluble thrombomodulin (rTM) in sepsis failed to show a 28-day all-cause mortality reduction [1]. Although the primary efficacy results did not support the administration of rTM, we found a positive signal in a post hoc analysis in the SCARLET trial. We also are concerned about the eligibility of patient selection possibly caused by a protocol amendment that lengthened the allowable time period from first qualifying INR until dosing as noted in the JAMA editorial [2]. In this trial, approximately 22% of subjects in the full analysis set (182/816 patients) did not fulfill the protocol-specified coagulopathy criteria (INR > 1.4 and platelet count > 30 × 10^9^/L) when the first dose of the study drug was administered. This population was thought to have a lower grade of coagulation disorder and/or lower disease severity. We have previously shown the importance of selecting a target population for anticoagulant therapy in sepsis that should be based on two critical components that include a “coagulation disorder” and “high disease severity” [3]. The inadequate population of 22% reported in JAMA may attenuate the power to detect the effectiveness of the intervention [1].

Recently, we reported the latest systematic review and meta-analysis [4] of recombinant thrombomodulin for sepsis including SCARLET trial results that were made public in August 2018. The data of five trials enrolling 1762 patients showed that the pooled estimate on mortality of recombinant thrombomodulin use was not statistically significant (risk ratio, 0.87; 95% confidence interval, 0.74–1.03; *P* = 0.10; *I*^2^ = 0%). A significant limitation of our meta-analysis was the lack of full results from the SCARLET trial. We therefore performed re-analyses by replacing the SCARLET results with a subgroup analysis of the proportion who still met the coagulopathy criteria at dosing. Consequently, mortality risk was reduced by the administration of recombinant thrombomodulin (risk ratio, 0.82; 95% confidence interval, 0.69–0.98; *P* = 0.03; *I*^2^ = 0%) (Fig. 1).

**Fig. 1:**
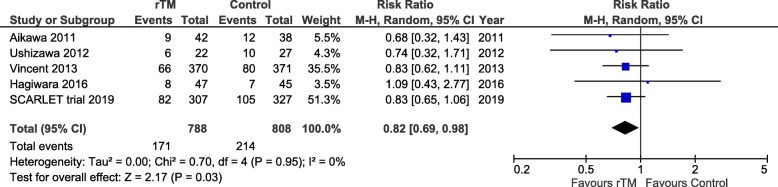
Forest plot of the comparison: rTM vs. control: all-cause mortality at 28 days. rTM, recombinant human thrombomodulin; M-H, Mantel-Haenszel; CI, confidence interval

Importantly, as a limitation mentioned by Vincent et al., post hoc analyses used in this re-analyzed meta-analysis were not planned a priori and need to be interpreted with caution. To properly implement precision medicine, a strategy of selecting the optimal target of an individual intervention is essential [5]. SCARLET is the first trial intended to examine the effects of anticoagulants in coagulopathic patients. We suggest that further trials of recombinant thrombomodulin should be performed that focus on the strictly eligible population that can potentially benefit from this therapy.

## Data Availability

Not applicable.

## Abbreviation

rTM: Recombinant human soluble thrombomodulin
